# Cultural Beliefs on Cleft lip and/or Cleft Palate and Their Implications on Management: A Systematic Review

**DOI:** 10.1177/10556656231209823

**Published:** 2023-10-30

**Authors:** H. Hasanuddin, Aisha AH. Al-Jamaei, Ellen M. Van Cann, Muhammad Ruslin, Marco N. Helder, Prasannasrinivas Deshpande, Tymour Forouzanfar

**Affiliations:** 1 Department of Oral and Maxillofacial Surgery/Oral Pathology, 1209 Amsterdam UMC Location VUMC, 1081 HV Amsterdam, The Netherlands; 2 Department of Oral and Maxillofacial Surgery, 4501 Leiden University Medical Center, Leiden, The Netherlands; 3 355664 Department of Oral and Maxillofacial Surgery Faculty of Dentistry, 64739 Hasanuddin University, Makassar, Indonesia; 4 Education and Teacher Training Faculty, Parahikma Institute of Indonesia, Gowa, Indonesia; 5 Department of Oral Medicine, Periodontology, and Radiology, Faculty of Dentistry, 108522 Sanaá University, Sanaá, Yemen; 6 Department of Head and Neck Surgical Oncology, UMC Utrecht Cancer Center, 8124 University Medical Center Utrecht, Utrecht, The Netherlands; 7 Department of Oral Medicine and Radiology, JSS Dental College & Hospital, 163766 JSS Academy of Higher Education and Research, Mysuru, Karnataka, India

**Keywords:** cleft lip, cleft palate, cultural beliefs, cleft causes, cleft responses, cleft managements

## Abstract

**Objective:**

This article examines and summarizes the published epidemiological literature on cross-cultural variations. Particular emphasis was put on addressing cross-cultural beliefs on the causes, management, and attitude toward cleft lip and/or cleft palate. A healthcare provider's awareness of these cross-cultural attitudes and beliefs is vital for promoting effective collaboration with patients’ families and ensuring a favorable medical outcome.

**Design:**

Systematic review.

**Setting:**

Not applicable.

**Participants:**

Patients with cleft lip and/or cleft palate, their families, their communities, and healthcare providers.

**Interventions:**

Not applicable.

**Main Outcome Measures:**

Not applicable.

**Results:**

All relevant and eligible studies were identified using PubMed and Google Scholar databases. The cultural belief was categorized and measured using Murdock's Theories of Illness. The study was reported in compliance with PRISMA guidelines. The quality of the selected studies was evaluated in accordance with the Critical Appraisal Skills Programme criteria (CASP). Fourteen articles covering thirteen countries on four continents met the inclusion criteria. In diverse communities, cleft lip and/or cleft palate was attributed to natural (infection, medication, improper diet, smoke, or radiation) or supernatural (God, eclipse, ancestral spirit, and curse) causes. Reported consequences include stigmatization, inappropriate treatments, leaving patients untreated, and infanticide.

**Conclusion:**

Cultural beliefs are the main cause of misconceptions surrounding a cleft lip and/or cleft palate. There is also a need for public health care providers’ intervention to educate society about the natural causes and ease of management, thereby increasing opportunities for patients substantially.

## Introduction

Cleft lip and/or cleft palate (CL/P) is typically considered one of the most frequent congenital malformations affecting orofacial areas. Worldwide, the incidence of CL/P varies considerably within multiethnic groups, with the highest prevalence reported among Asians and Native Americans (1 per 500 live births).^1^ CL/P could be one feature of more than 300 syndromes. Nonetheless, two-thirds of these conditions are non-syndromic (isolated), and their etiology is unknown so far.^2^

CL/P can be classified as complete or incomplete and unilateral or bilateral. Obviously, with the increasing severity of the cleft, there is an increase in levels of morbidity among children with CL/P. Of these morbidities, the children with CL/P could have obvious facial disfigurement, speech, hearing, feeding difficulties, and psychosocial challenges that can profoundly lower their life outcomes. Of note, the consequences of CL/P are not limited to the children, but their parents might also be severely affected. For example, parents of a child with CL/P have been noted to complain of psychological burdens, such as helplessness, guilt, distress, or depression.^3^ Another critical hurdle facing the parents is the burden of care because the patients require many surgical corrective therapies, special education, rehabilitation, and other nonmedical services.^4^ These physical, psychological, and financial burdens are often worsened by stigmatization, particularly in lower-income countries, where the causes of this phenomenon are often misunderstood, and people resort to their cultural perceptions to provide a plausible explanation for its occurrence. Indeed, linking these facial defects with cultural beliefs could further exacerbate the social stigma and exclusion and negatively affect the management protocol.

Historically, Ballantyne provided significant overviews describing the past theories suggested to explain the causes of deformities, including facial clefts in infants.^5^ Of particular interest is that maternal impression theory has been found to be culturally universal. Some recent works have also been conducted to describe the current cultural perceptions, ideas, or folklore surrounding deformity in specific contexts and settings across the globe.^6,7^ Such insights into various assumptions concerning cleft causation and management are valuable to enrich cross-cultural understanding of these conditions, mainly because the cultural background of the CL/P patients and their families can significantly influence their choices of medical and psychosocial treatments.

It is widely accepted that the systematic neglect of cultural beliefs in health and healthcare is a significant barrier to advancing the highest standard of health worldwide. Concerning CL/P patients treated by multidisciplinary clinical care, gaining knowledge on cross-cultural variation and attitudes toward these conditions is crucial to improving the medical outcomes and social development of these groups of patients. Even though several reports have evaluated the cultural attitude toward CL/P causation, management, and the reaction of societies in specific countries, to our knowledge, no systematic review of epidemiological literature has discussed and summarized the cross-cultural variations on these topics so far.

To achieve this aim, the study seeks to answer three specific questions: (1) what are the causes of CL/P from cultural perspectives, (2) what cultural medications are used to treat a CL/P patient, and (3) how is society around the patients respond to the birth of a CL/P child?

## Materials and Methods

This systematic review was conducted following the guidelines of the Preferred Reporting Items for Systematic Reviews and Meta-analysis (PRISMA) checklist.^8^ The Prisma Statement checklist is presented in the supplementary material (Tables S1 & S2). It was also registered at the International Prospective Register of Systematic Reviews (PROSPERO) database (CRD42022384382).^9^ Ethics approval and informed consent prior to the study were not required for this systematic review because the authors used publicly accessible documents as data and evidence.

### Inclusion Criteria

Studies were considered eligible if they aimed to assess the perspectives of the societies and/or parents on the cause, treatment, and response to CL/P. Given the variability in the definition of perspective in the literature, for this review, we defined perspective as follows: 1) Ideas and perception of the causes of CL/P, 2) Belief and perception of the causes of CL/P from both religious and cultural perspectives, 3) Knowledge about the etiology of CL/P, 4) Reaction to the birth of a child with CL/P, 5) The response toward the grown-up CL/P patients. 6) How a society treats CL/P patients, 7) Experience of patients and family regarding the issues surrounding CL/P, and 8) Miscellaneous aspects associated with CL/P. All measurements, including validated or invalidated questionnaires, interviews, and observations, were considered suitable. The included articles were English language sources only.

### Exclusion Criteria

Literature reviews were ineligible for data extraction, yet they were considered for cross-reference purposes. Studies that evaluated cultural perceptions on the causes, treatment, and responses to child deformities other than CL/P were also excluded.

### Information Sources and Study Selection

A thorough search was conducted to explore PubMed and Google Scholar to select the articles. All searches were performed in February 2020 and updated in March 2022. The Boolean operators “AND” and “OR” combined and narrowed the search. All variations of (cleft lip and/or palate, culture, religion, ideas, perspective, response, treatment, and belief or belief) were searched in combination with all other search terms (details in appendix). After determining the studies relevant to the research questions, the reference lists of those studies were searched as well for similar studies. There were no restrictions regarding the geographic origin or year of publication.

The first two authors (H&AAA) worked independently to find related literature and made a narrative database in Microsoft Excel, which included: a bibliographic record, type of source, title, setting, cultural perspective on the causes, management, and responses to the birth of a child with a CL/P. Further, a proforma was prepared by the same authors to identify the following features of each study: checked for duplication, checked if the sources met all the specified eligibility criteria, checked the data reliability and validity, and reviewed the narrative data that had been tabulated. These two authors also performed a quality assessment to prevent the inclusion of literature from unreliable sources. The entire selection process was monitored by further authors (TF & MNH)

As reported in the included literature, the cultural belief was categorized and measured using the causal attribution theory of Murdock et al.^10^ (Table 1). This category was used because it is the most comprehensive theory to explain illness causation. This category was made after thoroughly studying 139 cultural regions in Africa, East Asia, North and South America.

**Table 1.** The Causal Attribution of Illness (Murdock's Theories of Illness).

*Causation*	*Definition/Description*
*Natural causation*	
1. Infection	Invasion of the body by a harmful microorganism.
2. Stress	Exposure of the victim to either physical or psychic strain
3. Organic deterioration	The decline of physical capacity because of the age failure of a particular organ.
4. Accident	Suffering physical injury under the circumstances excludes both intentions on the victim's part and suspicion of supernatural intervention.
5. Overt human aggression	The willful infliction of bodily injuries on another human being
*Supernatural causation*	
6. Fate	Astrological influences, individual, predestination, or personified ill-luck
7. Ominous sensation	The experience of portent (not merely to portent illness)
8. Contagion	Contact with a polluting object, substance, or person
9. Mystical retribution	Act of violation of some taboo or moral inaction: food, sensory, sex, etiquette, ritual, property, and verbal
10. Soul loss	The temporary departure of the patient's soul from his body
11. Malevolent	The direct hostile, arbitrary, or punitive action of evil or affronted supernatural beings.
12. Sorcery	Aggressive use of a magical technique by a human being, either independently or with the assistance of a magician or shaman

### Quality Assessment

The quality of each included study was assessed independently by three authors (H, AAA&MNH) based on the criteria as formulated in the Critical Appraisal Skills Program (CASP) tool.^11^ Each item in the CASP checklist was assigned a numerical value: Yes = 1, Can't tell = 0, No = 0. The total score for each included study was calculated with a maximum possible score of nine. Articles with a score equal to or greater than 7 points were classified as having high quality, while studies that did not reach this score were considered lower quality. Any disagreement between the authors was resolved by discussion with a further author (MR &TF). The median score for the included studies was 8.6, showing that the articles included in this review are of high quality. The quality assessment results can be viewed in the supplementary material (Table S3).

## Results

### Literature Search

**Figure 1.**
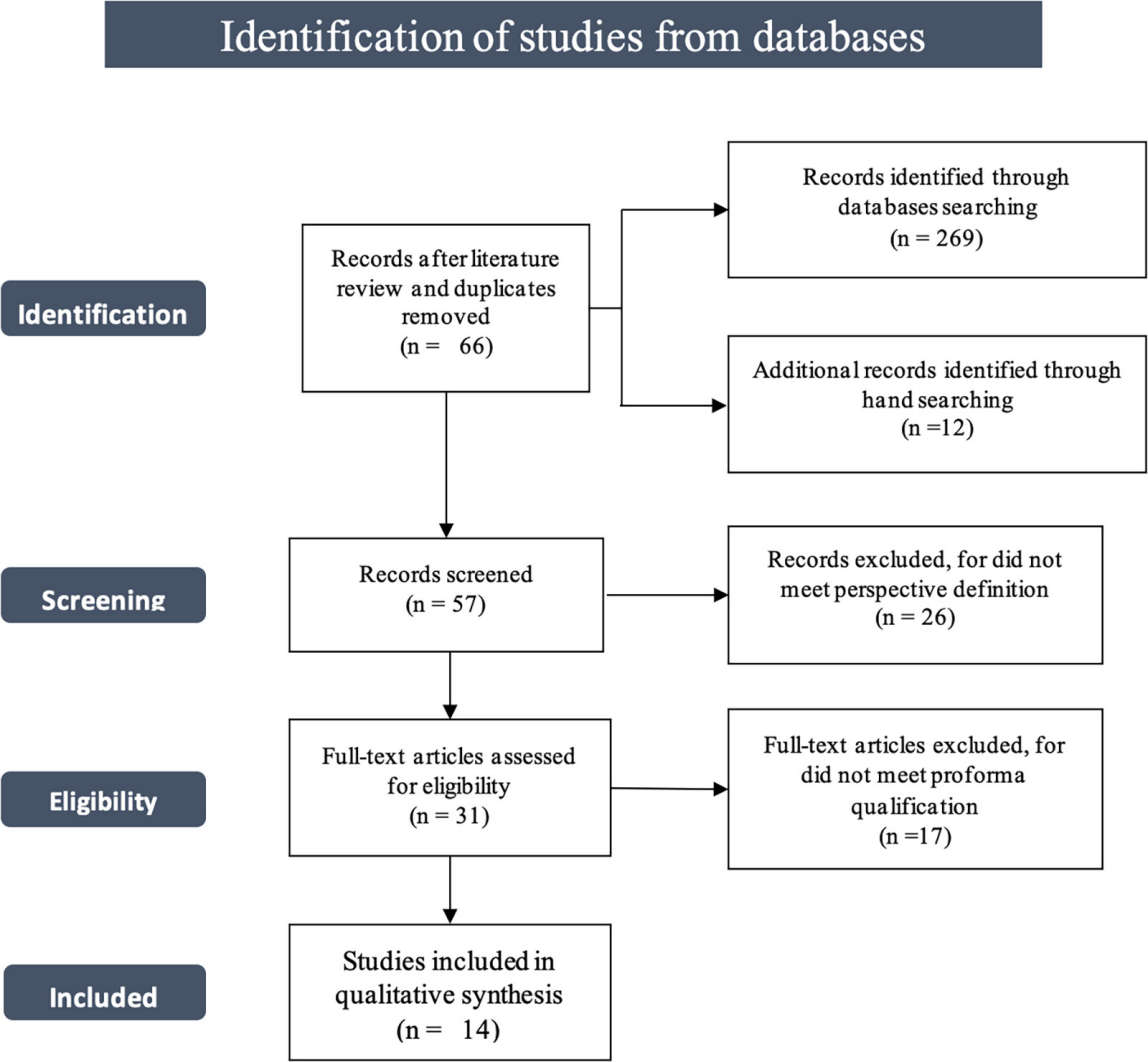
Flow diagram of the study selection process.

The selection process (Figure 1) fetched 269 PubMed and Google Scholar articles. Physical searching from the bibliography of the articles resulted in an additional 12 articles. After titles and abstract screening, 210 records were excluded as the papers did not include the perspective on CL/P's causes, treatments, and management of CL/P. Five articles were excluded due to duplication.

Among the 57 Full-text papers reviewed, 26 were excluded as they did not satisfy the inclusion criteria. Thirty-one papers fulfilled the inclusion criteria, and 17 were excluded due to a lack of quality assessment. Amongst the 17 excluded articles, one did not clearly state the beliefs of the causes and treatment of CL/P, nine book chapters referred to other sources, five articles’ full text were inaccessible, and the English version of two articles was not found. Finally, fourteen records were included in the final qualitative analysis.

### Description of Studies

**Figure 2.**
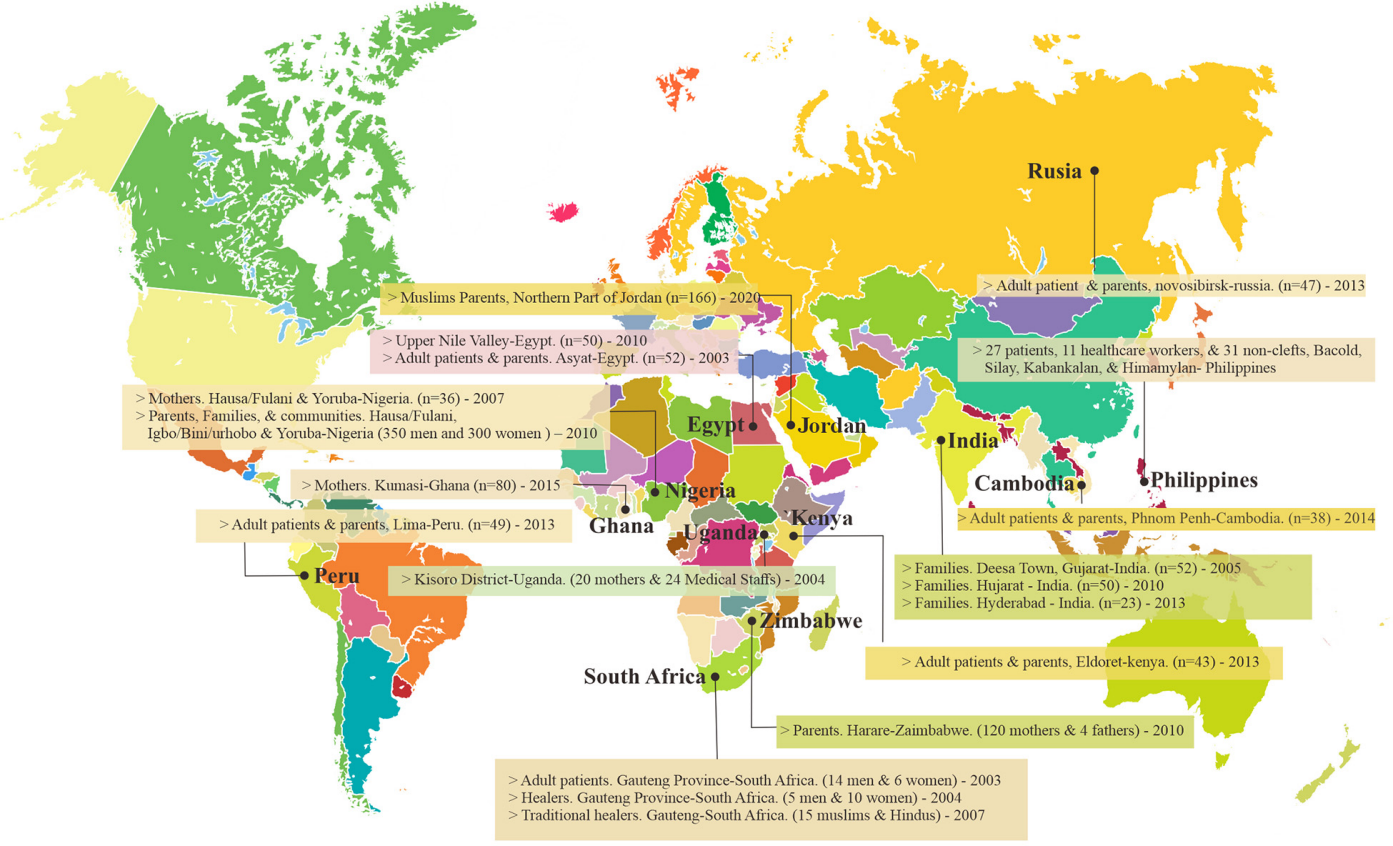
Countries of the studies included in the systematic review.

Table 2 shows the selected characteristics of all 14 included studies that were structured and categorized following the Murdock et al. typology. These studies were based in thirteen countries on four different continents: Africa (South Africa, Zimbabwe, Egypt, Nigeria, Kenya, Uganda, and Ghana), Asia (India, Philippines, Jordan, and Cambodia), Europe (Russia), and South America (Peru) (Figure 2). The geographic distribution of studies included in our review was heavily focused on African countries (n = 7, 53%). These studies estimated three essential topics on the ethnomedical aspect of CL/P, including the cultural beliefs of the causes, responses to birth, and management.

**Table 2.** Summary and Category of the Cultural Belief of the Causes, Responses, and Management of CL/P.

**Author(s) and year**	**Study aim**	**Research design**	**Data collection**	**Sample characteristics**	**Main study findings**				
					**Beliefs about the causes**	**Natural**	**Supernatural**	**Treatment**	**Response to the birth of CL/P child**
(Ross, 2003)^12^	To investigate how South African adults with repaired CL/P perceive their quality of life in relation to communication, education, employment, family and marital life, social life, and emotional problems.	Qualitative	Semi-structured interview	Adult patients, Gauteng Province, South Africa (14 men and 6 women).	Punishment from God.		Mystical retribution	Combined herbal and animal remedies.	*Not reported*
					An obstruction in the womb.	Infection			
					Inherited.	Organic deterioration			
					A random thing happened.		Fate		
					Predestined by God.		Fate		
					The mother played with a sharp object during an eclipse.		Mystical retribution		
					Medications.	Infection			
					A curse from a jealous lady.		Sorcery		
					A nurse accidentally cut the child's lip.	Accident			
					The child was dropped on the face after birth.	Accident			
					The mother spoke evil words.		Mystical retribution		
(Ross, 2004)^19^	To investigate the approaches of a group of traditional healers on the treatments of CL/P.	Qualitative	Semi-structured interview	Traditional healers who had been in practice for at least a year in Gauteng Province, South Africa (5 males and 10 females).	Curses of jealous people.	*Not reported*	Sorcery	Plant and animal products. Healing mental problems. Healers refer to Western doctors. Community concerns that Western doctors did not treat patients holistically. Traditional healers consult with Western doctors.	*Not reported*
					Ancestral spirits expressed their thirst for blood.		Ominous sensation		
					Mother was punished as she tried to steal another woman's child.		Mystical retribution		
					Ancestors cursed the baby because the family held a ritual in the wrong place.		Mystical retribution		
					The evil spirit possessed the baby.		Spirit aggression		
					It was attributed to bewitchment.		Spirit aggression		
(White, 2005)^6^	To determine the perceptions of parents regarding the causes of CL/P.	Qualitative	Semi-structured interview	Families of 52 patients (32 male and 20 female), in Deesa Town, Gujarat, India.	God's punishment for sins committed in a past life.		Mystical retribution	Education through the radio and television. Surgical treatments.	Five children were isolated, never leaving their homes and never attending school. One child was raised by grandparents after being abandoned by her parents.
					Genetics.	Organic deterioration			
					A curse of evil for past sins.		Mystical retribution		
					Mother looking at certain animals.		Mystical retribution		
					Mother consumed the wrong food during pregnancy.	Organic deterioration			
(Olasoji et al., 2007)^16^	To investigate CL/P patient mothers’ traditional beliefs regarding the cause of CL/P.	Qualitative	Interview	36 mothers of CL/P patients accompanied their children to two Nigerian teaching hospitals’ maxillofacial clinics. Nigerian Hausa/Fulani and Yoruba.	Punishment related to wrongdoing		Mystical retribution	Herbal, spiritual cleansing, prayer, ritual, and sacrifice. After giving them traditional treatment, they were referred to the hospital by the traditional healers.	The patient was left untreated as the Family reluctant to “Interfere with God's will.”
					God's will.		Fate		
					Curses from wicked people.		Sorcery		
					Pregnant women were going out during an eclipse.		Mystical retribution		
					Genetics.	Organic deterioration			
(Ross, 2007)^7^	To compare the perspectives of Muslim and Hindu traditional healers on the etiology and treatment of CL/P, the rationale for consulting them, and collaboration with Western professionals.	Qualitative	Interview	15 traditional Muslim healers and 8 traditional Hindu healers in Gauteng, South Africa	God sent.		Fate	Eastern medicine practitioners use prayer and faith in God, a psychological approach, and Ayurvedic medicine. Psychological support.	*Not reported*
					The mother handled a sharp object during an eclipse.		Mystical retribution		
					God's will.	*Not reported*	Fate		
					A pregnant woman drank/ate food during an eclipse.		Mystical retribution		
(Mzezewa, 2010)^14^	To explore the beliefs regarding the causes of CL/P to determine the attitudes of mothers and the general public toward CL/P babies.	Mixed methods	Structured questionnaire	120 mothers and 4 fathers of CL/P children presented for the first time to the plastic and reconstructive surgery outpatient clinic at Parirenyatwa University Teaching Hospital in Harare, Zimbabwe.	Witchcraft/ancestral spirit.		Witchcraft		Parents admitted taking the infant out and concealing baby-affected areas. CL/P child was stigmatized.
					Contraceptive pill.	Infection			
					Infidelity.		Mystical retribution		
					An act of God.		Fate		
(Oginni, et al., 2010)^15^	To determine the knowledge and cultural beliefs about the etiology and management of CL/P in Nigeria's major ethnic groups.	Mixed methods	Structured Questionnaire	Parents, family, and community (350 women and 300 men) Hausa/ Fulani, Igbo/Bini/Urhobo, and Yoruba, Nigeria.	Evil spirit/witchcraft.		Mystical retribution		
					Infection.	Infection		*Not reported*	Cleft malformation served a protective function against the high newborn mortality rate and should not be treated.
					Drugs/chemical products.	Infection			
					The mother attempted to do an abortion.	Accident			
					Abnormal gene.	Organic deterioration			
					Congenital hereditary.	Organic deterioration			
					Smoke.	Infection			
					Radiation.	Infection			
					Malnutrition.	Organic deterioration			
					Environment.	Infection			
					Parents’ sin.		Mystical retribution		
					God's Will.		Fate		
					God had run out of skin.		Mystical retribution		
(El-Shazly, et al., 2010)^20^	To identify parental perception regarding the causes of CL/P, belief systems that might affect these perceptions, societal reaction to the children, and the degree of social interaction.	Qualitative method	Interview	50 patients’ families were seeking care at Operation Smile missions in two different rural communities in Gujarat, India, and Upper Nile Valley, Egypt.	Punishment from God.		Mystical retribution	*Not reported*	Cleft should be left untreated, as treatment would interfere with God's will.
					Harmful food or drugs.	Infection			
					The use of intrauterine contraception.		Mystical retribution		
					Witchcraft.		Witchcraft		
					Mother gazed at a camel during pregnancy.		Mystical retribution		
					Genetic.	Organic deterioration			
					God's will.		Fate		
(Hirsch, 2010)^18^	To describe beliefs about the cause, prevention, and treatment of CL/P among working-class people and healthcare workers.	Qualitative	Interview and observation.	27 patients, 11 healthcare workers, and 31 non-clefts in Negros Occidental in Philippines (Bacolod, Silay, Kabankalan, and Himamylan)	An accident during the pregnancy.	Accident		*Not reported*	*Not reported*
					Environmental factors.	Infection			
					Force to the fetal face.	Accident			
					Inheritance.	Organic deterioration			
					Punishment for practicing witchcraft or prostitution.		Mystical retribution		
					The patients are regarded as evil spirits.		Mystical retribution		
					God's will.		Fate		
(Naram, et al., 2013) ^17^	To identify cultural beliefs regarding the cause and treatment of CL/P.	Qualitative	Semi-structured interview.	Family members of 23 CL/P patients awaiting surgery at the Gosla Srinivas Reddy Institute of Craniofacial Surgery (GSR) in Hyderabad, India.	A consanguineous marriage.	Organic deterioration		Burying the child on the abdomen or up to the head in the sand and exposing him or her to direct sunlight for a day.	The child was perceived as a sign of bad luck and had to be “gotten rid of.”
					Witchcraft.		Witchcraft		
					God's will.		Fate		
					Looking at a child with a facial deformity.		Mystical retribution		
					Infection.	Infection			
					Bad luck.		Mystical retribution		
					Self-blame.		Fate		
					Diet and medication.	Infection			
					Stress.	Stress			
					One's behavior.		Mystical retribution		
					Supernatural curse.		Sorcery		
					Influence from the previous life.		Mystical retribution		
					Medication.	Infection			
					Witchcraft.		Witchcraft		
					Bad omen.		Mystical retribution		
					Sins from a past life		Mystical retribution		
(Mednick, et al., 2013) ^21^	To describe and compare the causes of CL/P in different countries	Mixed methods	Interview and semi-structured questionnaire	Adult patients and their parents presented for evaluation and possible surgery in six developing nations during Operation Smile missions. 43 in Eldoret-Kenya, 47 in Novosibirsk-Russia, 38 in Phnom Penh-Cambodia, 50 in Deesa-India, 52 in Asyat-Egypt, and Lima-Peru. (n = 279)	Genetic.	Organic deterioration		*Not reported*	
(Kesande, et al., 2014)^13^	To determine the period prevalence, pattern, and perceptions of parents/guardians and health workers toward children with CL/P.	Qualitative	Review of medical records and interview	20 CL/P patient's medical records, interviews with 20 mothers who delivered babies with CL/P, and 24 Medical Staff interacted with mothers in Kisoro Hospital and St. Francis Hospital, Mutolere in Kisoro District-Uganda	Drugs	Infection		*Not reported*	In their respective communities, the children were not accepted. The mothers did not bring their children outside for fear that someone would see them and spread rumors.
					Bad omen		Fate		
					Environmental	Organic deterioration			
					Witchcraft		Mystical retribution		
					Genetics.	Organic deterioration			
(Antwi-Kusi, et al., 2015) ^22^	To evaluate the experience of CL/P patients’ mothers on etiology, family reaction, and treatment.	Mix methods	Structured questioner	80 mothers of CL/P children reporting at the Cleft Clinic of the Komfo Anokye Teaching Hospital in Kumasi, Ghana.	Superstitions.		Mystical retribution		6 nuclear family was avoided, 3 families were indifferent, and 70 were supportive.
					Punishment from God for committing a sin		Fate		
(Alfwaress, et al., 2020) ^30^	To determine the social and religious beliefs and behaviors toward children with CL/P.	Quantitative	Semi-structured questionnaire	166 Muslim parents from the Northern part of Jordan.	Evil Spirit		Mystical retribution	Parents indicated the possibility of seeking traditional medication.	*Not reported*

### Beliefs on the Cause of CL/P

Beliefs prevailing in societies concerned with the causes of CL/P vary significantly across the globe, most of which are linked to natural causes. Our results showed that infection was the most commonly held cultural belief regarding natural causes.^12,13^ In India, the infection was assumed to be caused by an obstruction in the womb or medication during pregnancy.^12^ Another study in India revealed that the wrong foods a mother consumed during pregnancy were a risk for CL/P.^6^ Medications were also suggested as a cause of CL/P in Zimbabwe, wherein the patients’ families believed that contraceptive pills taken by the mothers during pregnancy were a significant risk factor.^14^ Likewise, the major ethnic groups in Nigeria believed maternal smoking and exposure to radiation or other environmental substances were etiologies of CL/P.^15^

A second common natural cause assumed as a risk factor for CL/P by the societies was genetic abnormalities. Such a belief was reported by four studies based on data from African countries (South Africa, Nigeria, Egypt, and Uganda) and two studies based on data from Asia (India and Philippines).^6,12,13,15,16^ In India, another belief that has received particular focus was that men or women who married their close relatives (consanguineous marriage) were more likely to have babies with a CL/P.^17^

We also identified two studies that suggested accidents as a cause of CL/P. One study from South Africa assumed that nurses were responsible for CL/P, for whom they either accidentally cut the baby's lips or dropped the baby on the face, which eventually resulted in CL/P malformation.^12^ Similarly, a study from the Philippines reported that the fetal forces associated with a mother falling might result in a baby being born with a CL/P.^18^

Another important aspect of cultural beliefs of CL/P attributable causations, despite the advanced understanding of the natural world, was that people continue to report beliefs in supernatural phenomena. Indeed, population beliefs that CL/P occurred as a God's destiny were reported in South Africa, Nigeria, Zimbabwe, India, Kenya, Russia, Cambodia, Egypt, Peru, Uganda, and Ghana.^7,12,15–18^ This observation indicates that believing in God's destiny was culturally universal. Even though a CL/P child was perceived as the child of God in most studied societies, linking those children with bringing bad luck to societies was also common.

Another supernatural causation reported in association with CL/P was the ancestor spirit. This belief was informed by traditional healers around Gauteng Province-South Africa.^19^ The healers said that a baby was born with a CL/P as a sign that an ancestral spirit thirsted for blood or was cursed by an ancestor. Society around the CL/P patients in Deesa town, Gujarat, India, perceived that CL/P was a curse from an evil.^6^ Some factors were associated with this curse, such as parents’ sin,^15^ gazing at the camel, practicing prostitution,^20^ and looking at a child with a CL/P,^17^ sins of past life, or destructive behavior.^21^ Overall, it seems that societies often hold multiple causal beliefs simultaneously.

### Response to the Birth of a Child with CL/P

Eight studies (Table 2) reported the reaction of societies toward children with CL/P and revealed that those babies were intentionally isolated, stigmatized, or left untreated. This was reported in India, Nigeria, Uganda, Zimbabwe, and Ghana.^6,13–20,22^ In Uganda, social rejection of the child with CL/P and ashamed feelings of the parents with their babies’ condition were common reactions.^13^ Additionally, the babies born with a CL/P were not given a family name, but they were called “*ajok*” (female) or “*ojok*” (male), a local language that means “Satan”.^13^ In Zimbabwe, patients with facial deformities and their mothers experienced social stigma, and because of that those mothers were found to cry over their baby's condition at the initial stages. The mothers also admitted to taking the baby to postnatal clinics and ensuring they covered the clefting area.^14^

Rural Muslim and Hindu families in Gujarat in India and the upper Nile valley in Egypt believed that facial deformity was a punishment from God. Those families avoided surgeries because they were concerned that if the child's cleft was surgically treated, the further child would suffer the same defect, for interfering with God's will.^20^ Likewise, in Nigeria, there was resistance to repairing facial cleft malformations because these defects were considered to serve as a protective function against the high newborn mortality rate.^15^ Of note, in Hyderabad-India, a study found that the neighbors of CL/P individuals thought of them as bad luck and should be gotten rid of. Such a belief may directly threaten the patients’ lives. These highly adverse reactions toward CL/P patients were not constant as a belief that the CL/P was the “will of God (Allah)” encouraged some families in India and Egypt to accept the CL/P child and relieved the concern about the child's health and social life.^20^

### Management of CL/P

Of thirteen studies included in this review, only six mentioned the type of treatment CL/P patients received. Even though the patients had been surgically treated, traditional medications and other healing practices were also used. These traditional means of healing were widely practiced in Hindu and Muslim communities in India, for instance. In Hyderabad-India, parents treated the CL/P by burying the patients in sand up to the head and leaving them in direct sunlight for the entire day.^17^

The rural area communities in India also used prayer and faith in God and applied a psychological approach, herbs, special diets, and other unique health practices to cure CL/P.^7^ The treatments varied and depended on the healer and healthcare professionals consulted.^23^ Although traditional healers in South Africa accepted that the patients should be medically treated, they still had to apply conventional medication/healing, considering Western doctors did not treat patients physically and mentally.^19^ In Nigeria, the Hausa/Fulani ethnic group reported that the patients would be referred to the hospital by traditional healers after being given conventional treatments.^16^

## Discussion

This systematic review aimed to explore studies investigating cultural beliefs on causes, management, and reactions to the birth of children with CL/P worldwide. The results have demonstrated that belief about CL/P causation varies across countries, and societies often hold multiple causal beliefs simultaneously. Beliefs in natural causes, such as infection and genetic factors, and supernatural causes, like God's destiny, were almost culturally universal.^14,15,18^ Practicing spiritual interventions and traditional medication, leaving patients untreated, stigmatization or even infanticides were among commonly reported responses to CL/P patients.

A wide range of beliefs was found concerning the etiology of CL/P that in most countries are not grounded in empirical science. These beliefs, instead, are strongly influenced by sociocultural views. Culture itself, which is difficult to define, implies ideas, perceptions, and values that collectively constitute a way of life. It is unsurprising, therefore, that cultural values and worldviews substantially impact communities’ attitudes toward causal attributions and treatment of these deformities. Hammond-Tooke (1989) maintained that the definition of health issues in various ethnic groups was oriented toward traditions or folks’ models.^24^ Of note, three paradigms are commonly held across all cultures to explain the causality of illness: naturalistic, personalistic, and emotionalistic.^25^ The naturalistic theory attributes illness to loss of balance inside the body and is the most accepted theory in the Western world. Personalistic and emotionalistic theories which attribute disease occurrence, including CL/P, to supersitional power and feelings like anger or jealousy, respectively, are widely spread in low-income countries.

Since most of our studies were conducted in the middle- and low-income world, this might explain why we have found different cultural beliefs simultaneously in one setting; most were either personalistic and/or emotionalistic. In India, for example, some believed that CL/P happened because mothers saw the eclipse during pregnancy, and others mentioned that CL/P was a curse from a jealous person.^17^ In Africa, mainly in Nigeria, some ethnic groups like the Yoruba attribute the etiology of the cleft to supernatural forces (evil spirits and ancestral spirits), while the will of God was believed to be the dominant cause of CL/P among the Hausa/Fulani groups.^16^ Additionally, Patel et al. reported that some people from South Africa attributed the etiology of CL/P to mothers speaking harsh words.^12^ Thus, governments or notable organizations should put more effort in encouraging the acceptance of naturalistic theory within middle- and lower-income countries.

Notably, Winkelman (2009) reported that families generally have little faith in scientific medical explanations and utterly rely on cultural views to define the causes and treatment of the illness.^26^ Unfortunately, some of the cultural beliefs are misconceptions, conceive negative stigmas, and result in a “folks’ illness,” “imaginary disease,” or “psychosomatic illness” that affects patients’ well-being and quality of life.^27^ One example concerning CL/P is that South African adult patients asked a priest why they had this kind of deformity, and the priest responded that it was because their parents were sinners.^12^ Viewing this deformation as a punishment for a parent's sins could constitute a significant negative social and emotional well-being. Although most of the patients in the same study (16/20) expressed that the CL/P did not affect their social life, still, twelve patients still felt introverted, anxious, shy, sensitive, and moody.

It is well known that cultural belief affects conceptions concerning CL/P etiology and plays an important role in shaping ontological health systems. It is often reflected in varied methods; patients or their parents choose to treat this facial defect. Based on the Western medicine paradigm, the proper management of patients with CL/P is carried out by a multidisciplinary team involving maxillofacial surgeons, pediatricians, plastic surgeons, and others. Nonetheless, many populations in low-income countries still believe in Eastern medicine and tend to use prayers, faith, and traditional medicine to heal CL/P.^17^ In contrast, we have seen in this review that most families from low-income countries consult traditional healers concurrently or prefer Western approaches. However, of particular interest, a study from Nigeria found that some families reported their referral to modern medical practitioners by traditional healers.^16^ Likewise, two major religions in South Africa advise using Western medication. Still, they practice traditional healing during psychosocial consultation, using herbs as cures and attempting to treat patients through “supernatural powers.” This emphasizes the importance of collaboration between traditional healers and Western practitioners on culturally sensitive issues.

CL/P is usually associated with facial disfigurements, dental problems, difficulty in speech, hearing, feeding, and many other challenges. Such challenges result in various reactions from parents, families, friends, and whole societies towards these children. One of the shocking findings in this review was that a study reported a direct threat to the safety of children born with CL/P, and the main reason was the belief that those children brought bad luck to their entire communities and should be gotten rid of.^17^

Another important reaction to the CL/P defect that needs great attention was leaving the child without treatment.^15^ Some families believe that since the deformity was a gift from God, no intervention should be done. These observations, in particular, revealed how cultural beliefs may lead to very devastating consequences. Bullying, rejection, and social stigma have been found as common responses to CL/P patients, even at the time, from their families. Of note, a recent systematic review evaluated the impact of social stigma on a child with CL/P and found significant and remarkable negative outcomes. For example, some children drop out of education, could not reach their ideal employment, face economic burdens, and eventually lose productivity in their societies.^28^

Globalization and immigration continue to increase and building societies with very diverse cultures becomes inevitable everywhere in this world. This diversity mandates the medical team responsible for the management of CL/P patients to consider these patients’ cultural backgrounds, mainly because the cultural perspectives may pose barriers to treating these patients appropriately. It is noteworthy that good cross-cultural awareness by all medical team members would improve communication and foster trust between CL/P patients and their families and the health care providers. The importance of this cross-cultural understanding by healthcare providers of their patient's backgrounds ultimately helps to create and deliver culturally competent services to all CL/P patients, irrespective of their ethnicity or race.

To overcome the misconceptions surrounding etiology and society's reactions toward CL/P patients, a wide range of communication approaches could be adopted in middle- and low-income countries. This was identified to be a highly relevant approach for vaccination programs lately, and a recent review described several approaches to accomplish this.^29^ Some of these approaches should primarily be directed to the families, while others to health care workers. For example, health education to parents or mothers during home visits or antenatal clinic visits, flyers and posters in hospitals and clinics, and radio-, TV- or social media-based health promotion campaigns that explain the naturalistic theory about causes of CL/P could be important tools to improve the societal knowledge about CL/P. The engagement of traditional and religious leaders as advocates for these naturalistic theories would be another intervention that might work very well in these societies. Frequently providing brochures and fact sheets to healthcare providers to update and enrich their knowledge might facilitate and spur efforts to educate society and convey appropriate CL/P information.

This study should be understood in the context of some limitations, which may have introduced bias in our assessments. Firstly, some classical works presenting folk beliefs, myths, and historical perspectives of the causes were presented in a non-English language, and an English translation was not found. Therefore, the researchers found it difficult to explore its valuable information. Secondly, most studies included in this review were in rural areas with low socioeconomic status and little or no educational qualification. Thus, the samples relied on cultural and religious beliefs to determine the causes and treatment. These might be an undeniable cause of bias in the sources included. Due to more advanced access to media technology worldwide, people's perspectives within the research populations may have changed, even in rural areas. Hence, more explorations of the current perception, reaction, and treatment of CL/P should be undertaken.

## Conclusion and Recommendation for Practice

Cultural belief about CL/P causation varies across countries, and societies often hold multiple causal beliefs simultaneously. While the naturalistic theory is attributed to the illness being accepted mainly by Western nations, personalistic and emotionalistic theories are shared among the populace of the low-income world. The reaction of families and societies to a child born with CL/P might lead to devastating consequences. A good cross-cultural understanding of patients’ and their families’ backgrounds will create a better opportunity to deliver culturally competent health services to all CL/P patients worldwide.

## Supplemental Material

sj-docx-1-cpc-10.1177_10556656231209823 - Supplemental material for Cultural Beliefs on Cleft lip and/or Cleft Palate and Their Implications on Management: A Systematic Review Supplemental material, sj-docx-1-cpc-10.1177_10556656231209823 for Cultural Beliefs on Cleft lip and/or Cleft Palate and Their Implications on Management: A Systematic Review by H. Hasanuddin, Aisha AH. Al-Jamaei, Ellen M. Van Cann, Muhammad Ruslin, Marco N. Helder, Prasannasrinivas Deshpande and Tymour Forouzanfar in The Cleft Palate Craniofacial Journal

## Appendix

*Keywords*

To review cultural and religious beliefs, these keywords have been applied, culture, religion, cleft lip, cleft palate, perception, assumption, idea, and belief. Further, those mentioned keywords were formulated into some keywords formula using the Boolean operator “and” and “or”; Cleft- 42.255 articles, Cleft Lip 16.995 articles, Cleft Lip and Palate- 14.375 articles, Cleft Lip and Religion- 21 articles, Cleft Lip and Palate and Religion- 15 articles, Cleft Lip and Palate or Religion- 75.726 articles, Cleft Lip and Culture- 298 pieces, Cleft Lip or Culture- 973.954 articles, articles, Cleft Lip and Palate or Culture- 971.363 articles, Cleft Lip and Palate and Perspective- 269.
